# Child maltreatment in Canada: prevalence and gender differences among youth

**DOI:** 10.24095/hpcdp.46.2.02

**Published:** 2026-02

**Authors:** Britt McKinnon, Harriet L. MacMillan, Ashley Vandermorris, Katholiki Georgiades, Emma Nolan, Christina Catley, Isabelle Lvesque, Lil Tonmyr

**Affiliations:** 1 Family Violence Epidemiology Section, Centre for Surveillance and Applied Research, Health Promotion and Chronic Disease Prevention Branch, Public Health Agency of Canada, Ottawa,; 2 Department of Psychiatry & Behavioural Neurosciences, Department of Pediatrics, McMaster University, Hamilton, Ontario, Canada; 3 Division of Adolescent Medicine, Hospital for Sick Children, Toronto, Ontario, Canada; 4 Department of Paediatrics, University of Toronto, Toronto, Ontario, Canada; 5 Offord Centre for Child Studies, McMaster University, Hamilton, Ontario, Canada; 6 Queen’s University Belfast, Department of Psychology, Belfast, Northern Ireland; 7 Centre for Population Health Data, Social, Health and Labour Statistics Field, Statistics Canada, Ottawa, Ontario, Canada

**Keywords:** child maltreatment, family violence, youth, surveillance, epidemiology, surveys, Canada

## Abstract

This study presents the first Canadian self-reported estimates of child maltreatment (CM) from youth using data from 5256 participants aged 15 to 17 years in the 2023 Canadian Health Survey on Children and Youth. CM prevalence was high, particularly for emotional abuse (44.9%) and exposure to caregiver emotional intimate partner violence (39.4%). Females reported higher prevalence of sexual abuse (8.1% vs. 1.5%) and emotional abuse (52.2% vs. 35.4%) than males. Youth identifying as nonbinary or with a gender different from their sex assigned at birth reported the highest prevalence across all CM types, including 22.4% for sexual abuse and 83.7% for emotional abuse. These finding underscore the need for targeted research and policies that address structural determinants of gender-based disparities.

**Highlights**

- This study uses data from the 2023 Canadian Health Survey on Children and Youth to quantify child maltreatment among youth aged 15 to 17 years in Canada.
- Nearly half of youth reported emotional abuse; over one-third were exposed to emotional intimate partner violence among caregivers.
- Cisgender females reported higher prevalence of sexual abuse and emotional abuse than cisgender males.
- Gender-diverse youth experienced the highest prevalence across all maltreatment types.

## Introduction

Child maltreatment (CM), including neglect, exposure to intimate partner violence (IPV) involving parents/caregivers, and physical, sexual, and emotional abuse during childhood, can have immediate and lasting impacts. In the short term, CM may lead to physical injury, emotional and behavioural difficulties, and academic challenges.^1^,^2^ Over time, it increases the risk of mental health disorders, substance use, chronic illness, and poorer socioeconomic outcomes.^3^,^4^ These harms result in significant societal and economic costs.^5^

In Canada, CM prevalence estimates often rely on retrospective adult reports, which are prone to recall bias and may not reflect recent trends.^6^,^7^ Although youth self-reports share limitations of retrospective, cross-sectional studies, they offer a meaningful improvement by providing more direct and timely insights into CM experiences.^8^ This study presents national CM estimates based on youth self-reports, stratified by gender, including gender-diverse youth, to offer a more current and comprehensive picture. Disaggregating by gender diversity reveals disparities missed in binary analyses and highlights the unique experiences of gender-diverse youth.

## Methods

We analyzed data from the cross-sectional component of the 2023 Canadian Health Survey on Children and Youth (CHSCY), which sampled children and youth aged 1 to 17 years as of January 31, 2023.^9^ The sample was drawn from the Canadian Child Benefit file, which covers 98% of this age group living in the provinces; the survey excluded those in the territories, on First Nations reserves and other Indigenous settlements within the provinces, in foster care, or in institutional settings. The response rate for youth aged 12 to 17 years was 38.1%.

This descriptive study focussed on 5256 youth aged 15 to 17 years who completed the survey by electronic questionnaire or computer-assisted telephone interview and agreed to share their data with federal, provincial, and territorial health authorities.^9^ This was the only age group to self-report on CM. Youth were asked about five CM types: physical abuse (3items), sexual abuse (2 items), emotional abuse (1 item), neglect (1 item), and exposure to IPV (2 items, assessed separately as exposure to physical or emotional IPV), based on validated measures of CM (Table 1). Response options ranged from “never” to “more than 10 times,” and were dichotomized as any experience versus never. Reference persons (“any adult” for physical/sexual abuse; “parent/guardian” for the others) reflect standard measures and distinguish caregiver maltreatment from other abuse.

**Table 1:** Child maltreatment questions for youth aged 15 to 17 years, Canadian Health Survey on Children and Youth, Canada, 2023

Type of child maltreatment	Survey questions (Response options: never, 1 or 2 times, 3-5 times, 6-10 times, more than 10 times)
N/A	The next few questions are about things that may have happened to you at any time while growing up and might be hard for you to answer. Your responses are important whether or not you have had any of these experiences. Remember that all information provided is strictly confidential.
Physical abuse^a^	How many times did an adult slap you on the face, head or ears or hit or spank you with something hard to hurt you?
	How many times did an adult push, grab, shove or throw something at you to hurt you?
	How many times did an adult kick, bite, punch, choke, burn you, or physically attack you in some way?
Sexual abuse^a^	How many times did an adult force you or attempt to force you into any unwanted sexual activity, by threatening you, holding you down or hurting you in some way?
	How many times did an adult touch you against your will in any sexual way, such as anything from unwanted touching or grabbing, to kissing or fondling?
Exposure to physical IPV^a^	How many times did you see or hear any one of your parents, step-parents or guardians hit each other or another adult in your home?
Exposure to emotional IPV^b^	How many times did you see or hear any of your parents or caregivers say hurtful or mean things to each other or to another adult in your home?
Emotional abuse^b^	How many times did any one of your parents, step-parents or guardians say things that really hurt your feelings or made you feel like you were not wanted or loved?
Physical neglect^b^	How many times did your parents, step-parents or guardians not take care of your basic needs, such as keeping you clean or providing food or clothing?

**Source: **Canadian Health Survey on Children and Youth, 2023.^9^

**Abbreviation: **IPV, intimate partner violence. 

^a^ Adapted from the Childhood Experiences of Violence Questionnaire Short Form.^10^

^b^ Adapted from the US National Longitudinal Study of Adolescent to Adult Health.^11^

Sex at birth and current gender were assessed as separate questions, consistent with recommended two-step approaches to measuring gender identity in surveys.^12^ Free-text gender responses were recoded by Statistics Canada as male, female, or non-binary. While the two-step approach improves inclusivity, recoding of free-text responses may introduce misclassification. Gender is reported using the categories “male”, “female,” and “gender-diverse.” We acknowledge that “male” and “female” are typically sex categories;^13^ however, given how the survey data were collected, in this context they reflect self-identified gender. Youth whose current gender differed from their sex assigned at birth, or who were coded as non-binary, were grouped as “gender-diverse,” while “male” and “female” refer to cisgender youth. 

Prevalence ratios (PR) were computed by dividing survey-weighted proportions, and 95% confidence intervals (CI) were derived using 1000 bootstrap replicate weights. Males were the reference group due to consistently lower CM prevalence.^14^ We present crude PRs because our objective was descriptive and age-standardization produced virtually identical estimates.

Missing data on CM items ranged from 4.1% to 5.3% and were addressed using complete case analysis. All analyses were conducted in Stata, version 17 (StataCorp LLC., College Station, TX, USA). 

## Results

Among youth aged 15 to 17 years, 49.3% identified as female, 46.8% as male, and 3.9% as gender-diverse. CM prevalence was high, with emotional abuse (44.9%; 95% CI: 42.9– 46.9) and exposure to emotional IPV (39.4%; 95% CI: 37.4–41.4) most common. Physical abuse (22.3%; 95% CI: 20.6–24.0) was also frequently reported, while sexual abuse (5.3%; 95% CI: 4.5–6.1), physical neglect (5.2%; 95% CI: 4.5–6.0), and exposure to physical IPV (7.6%; 6.5–8.7) were less common. Prevalence varied by gender identity (Table 2), and estimates for gender-diverse youth had wider confidence intervals due to small numbers.

**Table 2:** Prevalence estimates and ratios for child maltreatment by gender, based on self-reports from youth aged 15 to 17 years, Canadian Health Survey on Children and Youth, Canada, 2023

Type of child maltreatment	Gender	Prevalence (95% CI)	Prevalence ratio (95% CI)
Physical abuse	Cis-male	20.3 (17.8–22.7)	1.0 (ref)
	Cis-female	23.3 (20.9–25.7)	1.15 (0.98–1.35)
	Gender diverse	37.5 (28.3–46.6)	1.85 (1.41–2.43)
Sexual abuse	Cis-male	1.5 (1.0–2.1)	1.0 (ref)
	Cis-female	8.1 (6.7–9.5)	5.24 (3.47–7.89)
	Gender diverse	22.4 (14.6–30.1)	14.49 (8.72–24.07)
Exposure to physical IPV	Cis-male	6.6 (5.1–8.1)	1.0 (ref)
	Cis-female	8.2 (6.6–9.7)	1.24 (0.92–1.66)
	Gender diverse	14.9 (8.4–21.4)	2.26 (1.38–3.69)
Physical neglect	Cis-male	3.9 (3.0–4.7)	1.0 (ref)
	Cis-female	5.8 (4.5–7.0)	1.49 (1.09–2.02)
	Gender diverse	17.9 (10.6–25.2)	4.60 (2.89–7.31)
Emotional abuse	Cis-male	35.4 (32.6–38.3)	1.0 (ref)
	Cis-female	52.2 (49.4–55.1)	1.47 (1.34–1.62)
	Gender diverse	83.7 (78.3–89.1)	2.36 (2.13–2.62)
Exposure to emotional IPV	Cis-male	33.5 (30.7–36.3)	1.0 (ref)
	Cis-female	44.1 (41.4–46.8)	1.32 (1.19–1.46)
	Gender diverse	60.7 (50.5–71.0)	1.82 (1.50–2.19)

**Source: **Canadian Health Survey on Children and Youth, 2023.^9^

**Abbreviations: **CI, confidence interval; IPV, intimate partner violence. 

**Notes: **Gender diverse includes youth who identify as nonbinary or whose gender differs from their sex assigned at birth (e.g. transgender). All child maltreatment questions are available in Table 1. 

Gender-diverse youth reported the highest prevalence across all types of CM. Sexual abuse was reported by 22.4% of gender-diverse youth (95% CI: 14.6–30.1), compared to 8.1% of females and 1.5% of males. Gender-diverse youth were 14.5 times more likely than males (PR=14.5, 95% CI: 8.7–24.1) and females over five times more likely than males (PR=5.2, 95% CI: 3.5–7.9) to report sexual abuse.

Emotional abuse was reported by 83.7% of gender-diverse youth, 52.2% of females, and 35.4% of males. Compared to males, emotional abuse was more than twice as prevalent among gender-diverse youth (PR = 2.4, 95% CI: 2.1–2.6) and was also more common among females (PR = 1.5, 95% CI: 1.3–1.6). Exposure to emotional IPV followed a similar pattern, with 60.7% of gender-diverse youth reporting this experience, compared to 44.1% of females and 33.5% of males (PR for gender diverse vs. male = 1.8, 95% CI: 1.5–2.2; PR for female vs. male = 1.3, 95% CI: 1.2–1.5).

Physical neglect was reported by 17.9% of gender-diverse youth, compared to 5.8% of females and 3.9% of males. Gender-diverse youth were 4.6 times more likely than males to report physical neglect (PR= 4.6, 95% CI: 2.9–7.3). Physical abuse was reported by 37.5% of gender-diverse youth, 23.3% of females, and 20.3% of males (PR for gender diverse vs. male = 1.8, 95% CI: 1.4–2.4; PR for female vs. male = 1.2, 95% CI: 1.0–1.4). Exposure to physical IPV showed a similar but smaller gradient: 14.9% of gender-diverse youth, 8.2% of females, and 6.6% of males (PR for gender diverse vs. male= 2.3, 95% CI: 1.4–3.7; PR for female vs. male = 1.2, 95% CI: 0.9–1.7).

## Discussion

Findings show a high prevalence of CM among Canadian youth aged 15 to 17 years, with notable gender differences. Gender-diverse youth reported the highest prevalence across all types, while females reported more sexual and emotional abuse than males.

Compared to CHSCY results, the 2023 Youth Risk Behavior Survey (YRBS), a US national survey of students (under age 18), showed a higher prevalence of physical abuse (31.8% vs. 22.3%), sexual abuse (7.1% vs. 5.3%), emotional abuse (61.5% vs. 44.9%), physical neglect (9.3% vs. 5.2%), and exposure to physical IPV (18.6% vs. 7.6%).^15^ Despite these differences, gender patterns were similar across surveys: females reported higher CM prevalence than males, with the largest gender gap in sexual abuse (USA: 11.8% vs. 2.7%; Canada: 8.1% vs. 1.5%).^15^ One exception was physical neglect, which was higher among US males, a pattern not observed in Canada. 

While the YRBS study did not report CM prevalence for gender-diverse students, our secondary analysis of the dataset^16^ (ages 15 to 17 years, using Swedo et al.^15^ measures) showed transgender and gender-questioning youth reported significantly higher CM prevalence than cisgender peers (see Table 3). Gender-diverse youth in the USA and Canada reported similar prevalence of sexual abuse (21.0% vs. 22.4%) and emotional abuse (89.1% vs. 83.7%), though physical abuse was higher in the USA (54.2% vs. 37.5%).

**Table 3:** Prevalence estimates for child maltreatment, based on self-reports from youth aged 15 to 17 years, National Youth Risk Behaviour Survey, United States, 2023

Type of child maltreatment	Gender	Prevalence (95% CI)
Physical abuse	Cis-male	29.1 (26.1–32.1)
	Cis-female	32.4 (29.8–35.0)
	Gender diverse	54.2 (49.3–59.1)
Sexual abuse	Cis-male	2.3 (1.4–3.2)
	Cis-female	10.7 (9.2–12.3)
	Gender diverse	21.0 (15.7–26.3)
Exposure to physical IPV	Cis-male	14.2 (12.1–16.3)
	Cis-female	22.8 (20.3–25.3)
	Gender diverse	30.1 (23.8–36.4)
Physical neglect	Cis-male	9.7 (7.6–11.7)
	Cis-female	8.6 (6.3–10.9)
	Gender diverse	8.4 (4.5–12.3)
Emotional abuse	Cis-male	53.2 (50.3–56.2)
	Cis-female	67.2 (64.1–70.4)
	Gender diverse	89.1 (85.0–93.3)

**Source: **National Youth Risk Behavior Survey 2023. 

**Abbreviations:** CI, confidence interval; IPV, intimate partner violence. 

**Notes: **Gender diverse includes youth who identify as transgender or gender-questioning. Child maltreatment measures as reported in Swedo et al.^15^ Data were analyzed by the authors using the publicly available dataset.^16^

Differences in CM prevalence between the USA and Canada may reflect both real variation in youths’ experiences (e.g., social and economic conditions, prevention/reporting systems, cultural norms around disclosure) and methodological differences (sampling approaches and survey design).^17^ While the CHSCY and YRBS use broadly similar CM measures, the CHSCY includes additional questions that distinguish between different experiences of sexual and physical abuse, whereas the YRBS groups multiple experiences into single items.^15^

Nationally representative estimates of CM among gender-diverse youth are scarce, particularly outside the USA, and the Australian Child Maltreatment Study is one of the few available examples.^18^ While its findings similarly indicate elevated prevalence among gender-minority youth, differences in age range (16 to 24 years) and measurement approach limit side-by-side comparison with the CHSCY.^18^ This underscores the need for additional population-based research, as well as work on protective factors, intervention strategies, and policies tailored to gender-diverse youth.^19^,^20^

***Strengths and limitations***

A strength of this study is its large national sample and use of validated CM measures. Using youth self-reports captures more recent maltreatment experiences compared to retrospective adult reports. However, despite these strengths, underreporting remains a concern, particularly for experiences like sexual abuse, due to stigma or fear of disclosure.^21^ Although adolescent reports reduce long‑term recall problems, self-reports can still be influenced by current mental health (e.g. depressive symptoms), but available evidence suggests such effects are generally modest.^22^ Despite weighting to reflect the national population, the CHSCY’s low response rate may still introduce nonresponse bias if CM is related to survey participation; some studies indicate health surveys may overrepresent participants with mental health challenges and underrepresent harder‑to‑reach groups, though findings are mixed and weighting cannot fully correct this.^23^,^24^ Finally, the exclusion of certain populations, such as youth in foster care and Indigenous communities, likely contributes to underestimation of national CM prevalence.^25^ Small numbers precluded separate estimates for transgender and nonbinary youth, as well as the examination of intersections of gender identity with sexual identity (e.g. lesbian, gay, bisexual).

## Conclusion

Our national, youth-reported estimates showed that nearly half of Canadian 15- to 17-year-olds experienced emotional abuse, and that gender-diverse youth experienced disproportionately high maltreatment across all types. To address these disparities, strengthening public health surveillance to explicitly capture gender identity would help build the evidence base needed to inform interventions for 2SLGBTQ+ youth.^26^ Surveillance efforts should also consider supplementing household sampling frames to include populations currently excluded (e.g. youth in foster care, many Indigenous communities). Future research should examine intersections of gender and sexual identities and differentiate within gender-diverse groups to better capture heterogeneity in experiences and needs. By integrating these data-driven, equity-focussed strategies, Canada can better address the lifelong mental and physical health costs associated with child maltreatment.

## Acknowledgements

We gratefully acknowledge Aime Campeau, Tracie Afifi, and Andrea Gonzalez for their instrumental roles in incorporating child maltreatment measures into the Canadian Health Survey on Children and Youth, and for their valuable insights into child maltreatment epidemiology in Canada. We also thank Carlos Tolentino from Statistics Canada for his support with analytical validation. This research did not receive any specific grant from funding agencies in the public, commercial, or not-for-profit sectors.

## Conflicts of interest

The authors have no conflicts of interest to declare.

## Authors’ contributions and statement

Conceptualization: LT, HM, BM; data collection and validation: IL, CC; data analysis: BM; methodology: LT, HM, BM, KG; writing—original draft: BM; writing—review and editing: LT, HM, KG, EN, AV, IL, CC, BM.

The content and views expressed in this article are those of the authors and do not necessarily reflect those of the Government of Canada.
