# Child maltreatment in Canada: prevalence and gender differences among youth

**DOI:** 10.24095/hpcdp.46.5.04

**Published:** 2026-05

**Authors:** Britt McKinnon, Harriet L. MacMillan, Ashley Vandermorris, Katholiki Georgiades, Emma Nolan, Christina Catley, Isabelle Lvesque, Lil Tonmyr

**Affiliations:** 1 Family Violence Epidemiology Section, Centre for Surveillance and Applied Research, Health Promotion and Chronic Disease Prevention Branch, Public Health Agency of Canada, Ottawa,; 2 Department of Psychiatry & Behavioural Neurosciences, Department of Pediatrics, McMaster University, Hamilton, Ontario, Canada; 3 Division of Adolescent Medicine, Hospital for Sick Children, Toronto, Ontario, Canada; 4 Department of Paediatrics, University of Toronto, Toronto, Ontario, Canada; 5 Offord Centre for Child Studies, McMaster University, Hamilton, Ontario, Canada; 6 Queen’s University Belfast, Department of Psychology, Belfast, Northern Ireland; 7 Centre for Population Health Data, Social, Health and Labour Statistics Field, Statistics Canada, Ottawa, Ontario, Canada

This corrigendum is being published to correct a co-author’s erroneous degree in the following article:

McKinnon B, MacMillan HL, Vandermorris A, Georgiades K, Nolan E, Catley C, Lvesque I, Tonmyr L. Child maltreatment in Canada: prevalence and gender differences among youth. Health Promot Chronic Dis Prev Can. 2026;46(2):61-6. https://doi.org/10.24095/hpcdp.46.2.02

**Before correction**

IsabelleLvesque,PhD^7^

**After correction**

IsabelleLvesque,MSc^7^

